# Facilitators influencing participation in digitally-based high-intensity interval training among individuals with axial spondyloarthritis - a qualitative study

**DOI:** 10.1186/s41927-025-00567-y

**Published:** 2025-09-10

**Authors:** Anna Torell, Emelie Wiking, Ingrid Larsson, M. Charlotte Olsson, Emma Haglund

**Affiliations:** 1 Department of Environment and Biosciences, School of Business, Innovation and Sustainability, Halmstad University, Halmstad, Sweden; 2 Department of Clinical Sciences, Section of Rheumatology, Lund, Sweden; 3 Department of Rehabilitation, Ängelholm Hospital, Ängelholm, Sweden; 4 Capio Movement, Department of Rheumatology, Halmstad, Sweden; 5 School of Health and Welfare, Halmstad University, Halmstad, Sweden; 6 Spenshult Research and Development Centre, Halmstad, Sweden; 7 Department of Health and Nursing, School of Health and Welfare, Halmstad University, Halmstad, Sweden

**Keywords:** Axial spondyloarthritis, Digital health, Exercise, High-intensity interval training, Qualitative research

## Abstract

**Background:**

Physical exercise is part of the recommended treatment for individuals with axial spondyloarthritis (axSpA). High-intensity interval training (HIIT) is an effective way to improve cardiovascular health without risk of increased disease activity. Nevertheless, there is a lack of knowledge regarding facilitating factors for digitally-based HIIT among individuals with axSpA.

**Objective:**

This study aimed to explore facilitators influencing participation in a twelve-week digitally-based HIIT intervention among individuals with axSpA.

**Methods:**

Individuals with axSpA (9 females/7 males) were recruited from the intervention group of a randomized controlled trial. The intervention included three high-intensity training sessions, two of which would be interval training, weekly for twelve weeks with digitally-based coaching by a physiotherapist. Individual semi-structured interviews were conducted with the participants after completing the intervention. At baseline, the median (min-max) age was 47 (28–65), disease activity (BASDAI) was 2.6 (0.2–5.5) and aerobic capacity was 31 ml O2/kg/min (24–54). A qualitative content analysis was used to bring out manifest content from the interviews. The analysis revealed three categories and eight sub-categories.

**Results:**

The participants described that HIIT was facilitated by intervention design, encouragement, and feelings of well-being. The intervention design facilitated HIIT to be feasible to perform, adaptable to everyday life, and by use of digital tools. The importance of inner drive, need for individual coaching, and support of social networks were highlighted as keys for encouragement. Feelings of well-being facilitated HIIT through perceived health effects and improved self-efficacy.

**Conclusion:**

A self-managed digitally-based HIIT intervention coached by a physiotherapist but performed outside the healthcare settings could be used as an additional non-pharmacological treatment for individuals with axSpA.

**Trial registration:**

Not applicable.

**Supplementary Information:**

The online version contains supplementary material available at 10.1186/s41927-025-00567-y.

## Introduction

Axial Spondyloarthritis (axSpA) is a chronic inflammatory disease characterized by inflammation in the spine and sacroiliac joints [1]. Symptoms such as pain, stiffness and fatigue can affect health-related quality of life and there is an increased risk for cardiovascular disease [2–4]. Recommended treatment is a combination of pharmacological and non- pharmacological interventions, including exercise [5].

Generally, a positive impact on cardiovascular morbidity and its risk factors is seen after aerobic exercise at different intensities, but is more evident at high intensities. High-intensity interval training (HIIT) involves repeated bouts of high-intensity exercise with recovery in between the intervals [6–8]. HIIT is shown to be effective for both healthy individuals and those with a chronic disease [6–8]. Moreover, results from previous studies have shown that individuals with axSpA can perform HIIT without worsening disease activity [9]. Performing exercise at high intensity is experienced as joyful but also a challenge, both physically and mentally, and it can be difficult to maintain adherence [10–13]. Experience of social support, professional guidance, and increased self-confidence have been shown to facilitate and contribute to adherence [11]. However, supervised exercise is resource-intensive; therefore, digital health has been introduced to healthcare, e.g., self- managed training with digitally-based support [14–17]. By exploring the individuals with axSpA´s perceptions, valuable insight can be gained on how they are physically and mentally impacted, and how healthcare providers can offer effective support when incorporating digitally-based HIIT into exercise routines. Thus, this study aimed to explore the facilitators influencing participation in a twelve-week digitally-based HIIT intervention among individuals with axSpA.

## Methods

### Design

This qualitative study has an exploratory design. Manifest qualitative content analysis with an inductive approach was used to explore facilitators influencing participation in a twelve-week digitally-based HIIT intervention. The study has been reported in accordance with the 32-item Consolidated Criteria for Reporting Qualitative Research (COREQ) checklist [18].

### Participants

The participants were recruited from a randomized control trial (RCT) (clinicaltrials.gov, no. NCT05781763) conducted in three rheumatology clinics in southern Sweden. Inclusion criteria for participating in the RCT were having an axSpA diagnosis, aged 18–65, unchanged medication in the preceding three months, and meeting the criteria for health-promoting physical activity [19]. Exclusion criteria were other underlying diseases, pregnancy, or already being on a HIIT routine. At the time of data collection, 16 participants were included in the intervention group in the RCT. They all completed the intervention and were asked about participating in the current study upon completion of the intervention. The participants were informed orally and in writing and signed an informed consent form before participation.

All 16 participants in the intervention group from the RCT agreed to participate and were interviewed. Self-reported information and assessment of aerobic capacity (VO2max) [20] at baseline of the RCT were used for describing the characteristics of the interviewed participants in the current study (Table 1). Health status was reported by EuroQol 5-Dimensions (EQ-5D), total score 0– 1, no health–full health [21]. Disease activity and physical function were reported by Bath Ankylosing Spondylitis Disease Activity and Functional Index (BASDAI and BASFI) [22, 23], with scores 0–10, best–worst. The median age was 47, health status (EQ-5D) 0.80, disease activity Bath Ankylosing Spondylitis Disease Activity Index (BASDAI) 2.6 and aerobic capacity 31 ml O2/kg/min (Table 1).

**Table 1:** Characteristics of interviewed participants, *n* = 16

	Baseline median (Range)
Female/Male (*n*)	9/7
Age (years)	47 (28–65)
Self-reported duration of illness (years)	10 (1–39)
VO2max (mlO_2_/kg/min)	31 (24–54)
EQ-5D health status (0–1)	0.80 (0.73–1.00)
BASDAI disease activity (0–10)	2.6 (0.2–5.5)
BASFI physical function (0–10)	1.0 (0.0-3.3)

Median (Range), EQ-5D = EuroQol health status, 0–1 (no health-full health) BASDAI = Bath ankylosing spondylitis disease activity index, BASFI = Bath Ankylosing Spondylitis Functional Index, 0–10 (best-worst)

The intervention in the RCT consisted of two HIIT sessions per week, reaching at least 85%–90% of maximum heart rate (HR) during the intervals, and one optional session with aerobic training, reaching at least 75% of maximum HR for 30 min. The coaching physiotherapists working at the clinics were initially instructed by one of the researchers on how to instruct HIIT. After one guided face-to-face session with a physiotherapist, the participants performed the training sessions independently, using a fitness watch as their digital tool for guidance (Polar Ignite, Polar Electro Oy, Kempele, Finland). The watch had three preset HIIT workout plans, 4 × 4 min, 5 × 3 min or 8 × 2 min, with 2–3 min´ active rest between the intervals. The participants could alternate between these workout plans, or equivalent ones, depending on their preferences. Their fitness watch was linked to an app that they downloaded on their smartphone to view their workouts. It was also linked to an online platform so the coaching physiotherapist could follow their training remotely. They received weekly individual coaching with the physiotherapist, conducted over the phone, and text message reminders three times a week. Two further guiding sessions, including individual face-to-face coaching with a physiotherapist, were offered if needed. The intervention is designed in a way that allows the structured training with HIIT to be combined with mobility and strength training according to the needs of each individual.

### Data collection

Data collection was conducted between August 2022 and August 2023. Individual semi-structured interviews, conducted by phone, featured open-ended questions such as “Can you describe how you experienced the training?” and “How did the high-intensity interval training affect you?”. Probing questions followed such as “Please, tell me more about…” and “What do you have in mind when you say…” to clarify and deepen the understanding of the participants´ experiences and thoughts e.g., facilitating factors. To ensure dependability, an interview guide was used (Additional file 1). Two pilot interviews were conducted to assess the interview guide, however, the open-ended questions remained unchanged, and these pilot interviews were included in the analysis. The interviews were carried out by two experienced qualitative researchers (IL, EH) with backgrounds in rheumatology, and no previous relationship with the participants. Each interview lasted 45–80 min, was audio recorded, transcribed verbatim, and pseudonymised. The total interview length was 13 h and 45 min.

### Data analysis

The first and second authors read the interviews several times, to become familiar with the content [24]. Sentences related to the study´s aim were extracted as meaning units, condensed and abstracted into codes. The codes were compared, based on similarities and differences, and grouped into eight sub-categories and three categories that reflected the central and manifest content of the interviews (Table 2). The research group, with experience in rheumatology and physiology, including physiotherapists (AT, EW, EH), a nurse (IL), and an exercise physiologist (CO), discussed the analysis until consensus was reached, thereby ensuring that the interpretation accurately reflected the data.

**Table 2:** Example of the analysis, from a meaning unit to a category

Meaning unit	Condensed meaning unit	Code	Sub–category	Category
**“**Yes, that has also been a motivation. Yes, it has. When they called from the clinic and asked how things had been, I could bring up things like having stomach pain and then I received support and help with that. That I should take a little break when my legs were hurting. That has also been supportive.” (participant 7)	Support through coaching facilitated and motivated completion	Coaching helps	Need for individual coaching	Encouragement facilitates HIIT

## Results

The results revealed that the intervention design, encouragement, and feelings of well-being facilitated participation in a digitally-based HIIT intervention among individuals with axSpA (Table 3). These three categories and the eight subcategories are described in detail in the text below. Selected quotations are presented to illustrate significant features of the categories. The number after each quotation refers to the specific participant.

**Table 3:** Categories and subcategories

Categories	Intervention design facilitates HIIT	Encouragement facilitates HIIT	Feelings of well-being facilitates HIIT
Subcategories	Feasible to perform	Importance of inner drive	Perceived health effects
	Adaptable to everyday life	Need for individual coaching	Improved self-efficacy
	Use of digital tools	Support of social network	

### Intervention design facilitates HIIT

The intervention design facilitated HIIT by making it feasible to perform in a way that was adaptable to everyday life, and through the use of digital tools.

#### Feasible to perform

The participants experienced that the intervention design facilitated HIIT because it offered the chance to perform HIIT in individually chosen activities and at preferred times and locations. This rendered it a feasible and easily accessible form of exercise to perform, e.g., running close to home directly after work. Depending on individual conditions and preferences, the participants conducted the HIIT sessions in various ways and could adjust their exercise routines according to their symptoms. The standardized structure and opportunity to independently plan their exercise were factors that made it seem feasible to perform. The design, with an initial leader-led session, and the chance to have additional training sessions, if needed, together with digital coaching once a week, created a sense of security. Even if the need for coaching was more important at the beginning of the intervention, the participants appreciated the coaching support throughout the period. On the other hand, the intervention design was also perceived as difficult to carry out in a practical sense, because they were instructed to reach and remain at a high intensity, and this influenced their choice of activity. The flexibility of the intervention design made it possible to perform the training both indoors and outdoors, which increased adherence. However, exercising outdoors could be challenging due to the fact that other road users, slopes, and wind affected the intensity. It appeared that the season and weather conditions influenced their motivation and choice of activity in both positive and negative ways. The HIIT sessions were experienced as physically and mentally demanding. Reaching a high HR was challenging.

*“It´s just that it´s tough. It´s really tough.” (participant 7)*

While participants found the intervention design feasible and effective for exercise, they expressed difficulty incorporating other forms, like mobility and strength training, into their routine alongside the intervention.

#### Adaptable to everyday life

The flexible arrangement and time efficiency of the design were described as crucial factors that rendered the intervention adaptable to everyday life, which facilitated HIIT. The participants appreciated being independent of others and being able to exercise when it suited them best. Since the HIIT sessions only took 40–50 min, the training was perceived as time efficient.

The participants appreciated the flexibility of being able to train near their home, workplace, or during their commute, rather than needing to go to a specific location. This was highlighted as a positive factor that contributed to the adaptability of the intervention design to everyday life.

*“For me*,* it´s been good to go out for a run because…you have your shoes at home and you just have to put them on and go out. You don´t have to go to any gym*,* you don´t have to book any group workouts*,* you don´t have to keep any appointments. You can really decide for yourself when you do it and for me … it is worth a lot.” (participant 13)*

Nevertheless, the participants experienced difficulties finding time for the intervention within their work-life balance. Finding strategies like planning for the exercise in advance, adapting it to everyday life, and creating a schedule for the coming week, were ways that ensured that the exercise would be carried out as planned.

#### Use of digital tools

The use of digital tools, e.g., a fitness watch and an app, was described as a crucial part of the intervention design which facilitated HIIT. Participants appreciated the ability to choose from three different pre-programmed workout plans. They found the workout plans challenging in different ways. The instant feedback from the fitness watch during the exercise and the data from the app were inspiring and motivated them to put in more effort.

*“As I said*,* the fitness watch is the reason that I´ve done what I´ve done.” (participant 15)*

However, they emphasized the need for ease of use, noting technical issues, such as difficulty reading the HR display or occasional loss of connection with the device, which they found frustrating and disruptive. The reminder text messages three times a week were described as helpful, but also sometimes annoying, as they were not synchronized with their personal schedules.

### Encouragement facilitates HIIT

The importance of inner drive, the need for individual coaching, and support from a social network were highlighted as encouragement that facilitated HIIT.

#### Importance of inner drive

Participants described HIIT as something to look forward to, to get physically exhausted from, and that it felt good to exercise at high intensity. They perceived that positive experiences that came with time were encouraging.

*”Now it´s time*,* now it´s the weekend and now one can go out into nature and train.” (participant 9)*

On the other hand, given that HIIT was experienced to be both physically and mentally challenging to carry out, and monotonous over time, the participants emphasized the importance of having inner drive; that is, having grit and being determined to carry out the exercise program.

#### Need for individual coaching

Participants described that regular individual coaching by a physiotherapist, both face-to-face and digitally contributed to encouragement which facilitated HIIT. The feedback was given both directly from the app and through phone calls. Receiving support and feedback on their training and results, as along with tools to carry out and overcome obstacles they encountered, also facilitated HIIT. The need for individual coaching was particularly important in the event of setbacks and difficulties; for example, when they had to resume training after a break related to illness or during periods of increased symptoms, e.g. pain, stiffness or fatigue. Through regular coaching, the perceived difficulties could be managed individually, for example with pain relief, by trying a different activity or by exercising at a different time of day. Setting specific goals together with the physiotherapist, to complete these three sessions per week, was highlighted as an encouragement factor that facilitated adherence to the intervention. The individual coaching gave the participants the feeling of being seen and cared for. Having someone who believed in their capacity to successfully complete the intervention was an encouraging factor that facilitated HIIT.

*“If there is a problem*,* you get tips and advice on what you can do to address it*,* and that´s incredibly valuable because then you can give it a try. And it´s the encouragement*,* the feeling that there is someone who believes in you*,* that I find very important. It contributes to a sense of* success.” (participant 5)

The fact that the coaching was carried out remotely and adapted to the participants´ needs facilitated regular contact, although it emerged that, in some cases, physical presence of a physiotherapist would have been desired.

#### Support of social network

The support from the participants´ social networks was perceived to be an important part of the encouragement that facilitated HIIT. Participants described the importance of support from those close to them, usually family members.

*“It´s like this now*,* I have my husband and he has been on me like a ferret*,* saying*,* ´No*,* come on*,* now you should train*,* you know.´ He hasn’t cheered me on like the coach did; instead he has made sure that I actually did what I was supposed to do.” (participant 5)*

It could be about reminders and reassurances to dedicate time to the training; for instance, partners taking care of children during training. The participants felt that the intervention design allowed participants to choose to exercise with others, if desired. This created new forms of social activity, which contributed how encouragement facilitated HIIT. On the other hand, participants expressed that being able to carry out the exercise on their own without having to consider others facilitated HIIT. Another aspect was that just knowing that others were doing the same provided a sense of social support, even at a distance. However, there were also expressions of a lack of social support, and that group sessions or a physiotherapist´s presence, in addition to the digitally-based support, would have facilitated HIIT through face-to-face encouragement.

### Feelings of well-being facilitate HIIT

The participants experienced that HIIT was facilitated by their feelings of increased well- being, in terms of both physically and mentally perceived health effects, and by improved self-efficacy.

#### Perceived health effects

The participants experienced positive physical health effects, such as improved fitness, increased energy, and better sleep quality. They also experienced improved mental health, such as increased stress tolerance and enhanced mood. Together, these positive feelings of well-being constituted a major factor in the facilitation of HIIT. In addition, the HIIT intervention allowed them to have some much appreciated “me-time”, when they could focus only on themselves and their health, and this contributed to the fact that feelings of well-being facilitated HIIT. Additional perceived health effects were change in pain experiences, in particular decreased pain, no flare-ups during the intervention, and reduced need for medication.

*“I could be completely drained*,* and I am after every session*,* but still*,* I gain so much*,* much*,* much more energy. Plus*,* I´ve had less pain. I find it easier to get up in the morning. Otherwise*,* it´s a significant issue with this disease – I used to have a lot of pain in the morning when I tried to get up*,* and generally*,* I was stiff at night. But I feel that it has improved. I’ve felt more agile in my body.” (participant 10)*

In contrast, some experienced increased pain which they perceived was linked to the exercise, such as back pain and periostitis. Nevertheless, adherence to the intervention remained unhindered, owing to the flexibility in the intervention design, which enabled them to identify alternative approaches to maintaining adherence, despite the presence of pain, on their own or with the digitally-based support of the physiotherapists. Participants experienced that this intervention led to a healthier diet and, for some, increased food intake. There were feelings of disappointment at the lack of weight loss. The risk that strict training with fast results in terms of fitness levels could easily transition into an unhealthy fixation was also brought to our attention.

#### Improved self-efficacy

Experiences of well-being facilitated HIIT through improved self-efficacy. The self-efficacy was improved by increased knowledge, both theoretical and practical, gained through their own positive experiences. The participants stated that using the fitness watch and the app, and seeing how their HR was affected by the activities, gave them new valuable insights into the type of activities that were suitable, and the level of effort required. Through regular digitally- based coaching, they learned how to adapt HIIT to accommodate any symptoms experienced. They expressed feelings of satisfaction after completing a training session, in practical terms as well as physically. Improved results, along with overcoming the physical challenges and their concerns about HIIT, increased their self-efficacy. This, in turn, facilitated adherence to the intervention.

*“It was interesting because*,* in previous years when I´ve trained*,* I probably haven´t even reached those heart rates*,* always staying around 165 or 80–85% of my maximum heart rate. So*,* it was fun to see that you could*,* and that there´s no need to be afraid of reaching those levels.” (participant 3)*

They also expressed that they had gained increased knowledge about how exercise influences their overall well-being, and that they can benefit from these experiences in other forms of training. In contrast, there were expressions of disappointment, failure, and frustration, when things did not go as planned. This could involve practical difficulties or having to control disease symptoms, such as pain.

## Discussion

The results of this qualitative study show that individuals with axSpA experienced that a twelve-week digitally-based HIIT intervention was facilitated by its design, encouragement, and experiences of increased well-being. The intervention design was feasible to perform and adaptable to everyday life. The use of digital tools, was highlighted as an important factor in facilitating HIIT. Inner drive, individual coaching by a physiotherapist, and support of social networks were important elements of encouragement. Perceived health effects and improved self-efficacy created feelings of well-being that facilitated HIIT. The results show that the intervention was feasible to perform and was facilitated by its flexible design, and it is important to have knowledge of these facilitating factors for the different members of the health team. Our results align with previous research, which reported that supervised HIIT interventions can yield positive experiences and be a viable method for individuals with axSpA [9, 11]. Compared to moderate-intensity continuous training, HIIT is reported to be more enjoyable, which enhances the likelihood of sustaining the routine over time [10, 25]. It is recommended that various activities be employed, to increase enjoyment and decrease the risk of musculoskeletal discomfort during performance, which also might contribute to adherence over time [10, 13]. Nevertheless, in this current study, the participants described that they missed other forms of exercising, e.g., strength and mobility training which are important experiences [26]. In clinical practice, it is important also to provide the opportunity to include these forms of exercise. The results show that HIIT is experienced as tough yet feasible to perform, which is in accordance with previous research [11, 27]. To achieve the best effect on cardiorespiratory fitness, it is recommended that, during the intervals, the HR reach 85–95% of maximum HR, corresponding to 17–18 on the Borg rating scale [7]. The discomfort associated with that high intensity can be relieved by keeping the intensity below 90% of maximum HR, which may be something to keep in mind, as this could influence adherence [25]. In this study, the intervention was described as time efficient and adaptable to everyday life, which promoted adherence. Even so, several participants pointed out difficulties fitting the exercise into their everyday lives. This is in line with the results of a previous study, which showed that one of the three top barriers of vigorous cardiorespiratory training was difficulties in incorporating exercise into the daily routine [12]. According to the inclusion criteria, all participants in this present study were physically active, according to the World Health Organization´s (WHO) recommendation, at baseline. Thus, in theory, the time spent exercising would not be more time-consuming. However, the level of physical activity was self-reported, which differs from objectively measured physical activity [28]. The results of the current study revealed that using a fitness watch was crucial, to be able to cope with tough training, and that user-friendliness is important. Using digital tools as part of healthcare is already implemented in various ways, and studies show good results after home-based exercise programs with digital support [17, 29, 30, 31]. This has also been shown for HIIT [14]. Participants described that ease of reading the fitness watch was important in order to reach and maintain an accurate HR during the sessions, even though they could use rating of perceived exertion (RPE) (6–20 scale) [32] as a complement. It has been shown that using RPE could be an alternative to a fitness watch [14]. Also, if the maximum effort is not achieved when performing the maximum HR test at baseline, the recommended HR levels will be inaccurate. Using both objective HR and perceived exertion could be an option, to ensure the correct intensity and reduce dependence on digital tools during exercise.

Inner drive emerged as an important quality to cope with regular HIIT. Experiencing HIIT as challenging and demanding is consistent with previous research [11]. Motivation is prominent among barriers to and facilitators of performing high-intensity training, especially among physically inactive individuals with axSpA [12]. This might indicate that, as a part of rehabilitation, HIIT is most suitable for those who already have an inner drive. Participants clearly expressed the need for individual coaching. They felt well-supported by the coaching physiotherapists and felt secure in their exercise during the intervention period, even though they only met the physiotherapist in person occasionally. They pointed out that having made an agreement together motivated them to adhere. This need for individual coaching, including goal setting, is shown to be of importance in behaviour-change interventions [33]. Participants found starting or resuming the intervention after breaks due to illness particularly challenging. Therefore, continuous coaching was essential to minimize breaks; however, the mode of contact – whether physical or digital – was not crucial. Previous research shows that distance contacts serve their purpose well. Digital coaching can enhance accessibility and promote greater equality, regardless of proximity to the nearest healthcare facility [34]. The support of social networks, mostly family and friends, was highlighted as an important element of the encouragement that facilitated HIIT. Previous research indicates the importance of including one´s social network to cope with a behavioural change [33]. The results of the current study reveal that the participants emphasized the flexibility of the design as a prerequisite for adherence. At the same time, some mentioned the lack of supportive and social context, which is perceived as a facilitating factor in adhering to HIIT [11, 27]. There is a paradox in the desire for support of social networks – the freedom of self-monitored exercise, versus a social setting with the guidance of a coach. Considering that axSpA is a chronic disease with intermittent flare-ups, individual preferences and the need for the support of one´s social networks may vary over time and need to be addressed individually.

The results show that feelings of well-being promoted HIIT through perceived and measured health effects. These findings are similar to previous research describing that HIIT can positively affect physiological and mental health [11, 35]. Experiencing good results of HIIT on physical fitness has previously been shown to increase motivation for HIIT among young adults [36]. In the present study, the participants experienced both decreased and increased pain, but still adhered to the intervention without hindrance, probably due to the flexible intervention design. Research has previously shown that disease symptoms can be a barrier to exercise among individuals with axSpA; however, it can also be a facilitator, especially for those who already are physically active [12, 37, 38, 39]. The results indicate that, by learning about themselves and about exercise, the participants´ self-efficacy was improved, which promoted HIIT. Participants experienced that, prior to the intervention, HIIT felt intimidating. They expressed previously having a cautious approach to exercise, to avoid pain. By participating, the participants discovered they could handle much tougher training intensities than anticipated. This newfound self-efficacy is confirmed by previous research [11, 27]. This experience can be applied to other forms of exercise, likely resulting in a more varied workout routine in training intensity. Participants highlighted the risk of negative effects on self- confidence and uncertainty, if they struggled with or could not complete the intervention. This is important to keep in mind when implementing HIIT, because mental barriers as lack of motivation, self-efficacy, familiarity with exercise and lack of social support have been found to be more evident than physical barriers to exercise [40].

### Strengths and limitations

Qualitative content analysis was chosen to reveal the variation and diversity in the material [24]. The dependability was strengthened by using an interview guide assessed through two pilot interviews. The participants were encouraged to express themselves openly and elaborate on their experiences, and the interviewers had broad competencies in rheumatology. The interviews were conducted over the phone, which could be both a limitation and a strength [34]. Credibility was strengthened by the authors regularly discussing data interpretation until a consensus was reached. Confirmability was ensured by using quotations to illustrate participants´ experiences. Transferability was ensured by recruiting a heterogeneous group in terms of sex and age, and from three clinics. The detailed description of the study context and participant characteristics allows readers to evaluate the applicability of the findings to other settings or populations. A limitation was that participants were recruited from a physically active subgroup of individuals with axSpA and were informed about the intervention design before agreeing to participate. This may mean that the included participants were more capable of taking on tough challenges and motivated to exercise, which could affect the transferability to the general axSpA population.

## Conclusion

According to the results of this qualitative study individuals with axSpA experienced that a self-managed digitally-based intervention with HIIT was facilitated by intervention design, encouragement, and increased well-being. The intervention design was feasible to perform and adaptable to everyday life. Utilising digital health by the use of digital tools was described as an important part of the design that facilitated implementation. Inner drive, individual coaching by a physiotherapist, and social network support were important elements of encouragement. Feelings of well-being facilitated HIIT through perceived health effects and improved self-efficacy. These facilitating factors are important to be aware of for different team members in the management of individuals with axSpA. The findings also indicate that a self-managed digitally-based HIIT intervention, coached by a physiotherapist but performed outside healthcare settings, could be used as an additional non-pharmacological treatment for individuals with axSpA. Future studies are needed to get more knowledge about the physiological, inflammatory, and self-reported effects of digitally-based HIIT as well as to investigate long-term experience and adherence to digitally-based HIIT.

## Supplementary Information

Below is the link to the electronic supplementary material.

Supplementary Material 1: Additional file 1, pdf: Interview guide

## Data Availability

The data supporting the results reported in the manuscript is stored on a security-protected server at Halmstad University and is not publicly available due to the ethical approval from the Swedish Ethical Review Authority. A code key is necessary for identification and only the researchers involved in the project have access to the code. The results will only be presented at group level.
