# Supplementary material for: Facilitators influencing participation in digitally-based high-intensity interval training among individuals with axial spondyloarthritis - a qualitative study
Source: BMC Rheumatol. 2025 Sep 10;9:104. doi: 10.1186/s41927-025-00567-y (PMC12421769; doi:10.1186/s41927-025-00567-y)
Supplement: Supplementary file 1 — Supplementary Material 1: Additional file 1, pdf: Interview guide [file 41927_2025_567_MOESM1_ESM.pdf]

Interview guide

1. Can you tell me about the exercise training you did during the project?
2. Can you describe how you experienced the training?
3. How did the high-intensity interval training affect you?
   - What positive effects did you feel the training had immediately and over time?
   - What negative effects did you feel the training had, immediately and over time?
4. Can you describe how you feel exercise affects your well-being?
5. What are your positive, and negative experiences with high-intensity interval training?
6. What barriers do you feel exist around high-intensity interval training?
7. What opportunities do you see with high-intensity interval training?
8. What barriers and opportunities do you feel exist for continuing high-intensity interval training after the end of the training period?
9. What factors have motivated you to do high-intensity interval training and what factors have limited you?
10. What are your thoughts on high-intensity interval training as part of your treatment?
11. What type of training would you choose to feel your best? Why?

Probing questions:

Please, tell me more about…

What do you have in mind when you say..?

What do you mean when you say…?
